# Effectiveness and safety of dietary supplements in the adjunctive treatment of psoriasis: a systematic review and network meta-analysis

**DOI:** 10.3389/fnut.2025.1718828

**Published:** 2025-12-11

**Authors:** Danping Chen, Jie Yang, Wenwen Yang, Xinying Liu, Zhihong Li

**Affiliations:** 1 Graduate School of Heilongjiang University of Chinese Medicine, Harbin, China; 2 The Fourth Affiliated Hospital of Heilongjiang University of Chinese Medicine, Harbin, China; 3 Graduate School of Tianjin University of Traditional Chinese Medicine, Tianjin, China

**Keywords:** psoriasis, dietary supplements, vitamin D, XP-828L, curcumin, network meta-analysis, systematic review, interleukins

## Abstract

**Background:**

Psoriasis, a chronic immune-mediated inflammatory skin disease, significantly impairs quality of life. Conventional treatments often pose safety risks or lack long-term efficacy. Dietary supplements show immunomodulatory and anti-inflammatory effects, but their adjunctive role in plaque psoriasis lacks a comprehensive comparison.

**Methods:**

A network meta-analysis (NMA) of 21 randomized controlled trials (RCTs), involving 1,463 patients with plaque psoriasis, was conducted using eight international and Chinese databases up to March 3, 2025. Interventions included vitamin D, XP-828L, fish oil, selenium, probiotics, curcumin, and micronutrients. Primary outcomes were Psoriasis Area and Severity Index (PASI), Dermatology Life Quality Index (DLQI), physician global assessment (PGA), interleukins (IL-6, IL-17, IL-23, IL-22), and adverse events. A frequentist NMA in Stata 17.0 used surface under the cumulative ranking curve (SUCRA) to rank efficacy and safety.

**Results:**

In 21 RCTs (*n* = 1,463), vitamin D supplementation significantly reduced PASI scores (mean difference = −3.29, 95% confidence interval [CI] − 6.38 to −0.20). XP-828L showed the highest probability of improving DLQI/PGA, and vitamin D + NB-UVB most consistently lowered IL-6/IL-17/IL-23; curcumin reduced IL-22. Adverse events were comparable across interventions (risk ratio 1.02, 95% CI 0.94–1.10). No supplement dominated across all outcomes, and overall certainty was low-to-moderate due to heterogeneity and imprecision.

**Conclusion:**

Dietary supplements may provide complementary benefits in plaque psoriasis; however, effect estimates vary by outcome, and certainty is low to moderate. Personalization is advisable, and confirmatory, larger RCTs with standardized dosing and longer follow-up are warranted. (Word count: 223).

**Systematic review registration:**

The review was registered at INPLASY (INPLASY202570119; DOI:10.37766/inplasy2025.7.0119).

## 1 Introduction

Plaque psoriasis is a common, chronic immune-mediated skin disease (1) characterized by well-defined erythematous plaques with scale, pruritus, and pain (2). Epidemiologic data suggest that lifestyle factors—such as chronic stress, sleep disruption, and poor diet quality—may precipitate onset or flares (3, 4). Recent evidence further links dietary patterns with psoriasis activity and severity, underscoring diet as a potentially modifiable risk factor (5). Prevalence varies across regions and ancestries, being lower in many Asian populations and higher in Europe and North America, reflecting genetic and environmental determinants (6). As a systemic immune disorder, psoriasis is associated with metabolic comorbidities and cardiovascular disease (7). Seasonal variation, notably during winter–spring exacerbations, is well documented, whereas epigenetic contributions remain hypothesized based on emerging mechanistic work (8). The chronic and recurrent nature of psoriasis imposes both physical and psychosocial burdens; anxiety, depression, and impaired quality of life are common, underscoring the relevance of DLQI outcomes (9). Unlike previous reviews focused on single supplements or pairwise comparisons, this study integrates multiple dietary supplements and outcomes (disease severity, quality of life, cytokines, and safety) within a unified network framework, offering a broader and more comprehensive synthesis.

Current therapies include (i) topical vitamin D analogs and corticosteroids; (ii) phototherapy and conventional systemic agents (e.g., methotrexate, cyclosporine); and (iii) biologics targeting TNF-*α*, IL-17, or IL-23 (10). Although effective, these modalities carry adverse effects and adherence considerations, and drug survival may be limited in routine practice (11), leaving several clinical needs unmet. These observations align with real-world adherence and long-term effectiveness/tolerability syntheses and support integrating patient preferences in chronic management (9).

Potential adjunctive options include omega-3 fatty acids, vitamin D, probiotics, curcumin, selenium, micronutrients, and XP-828L. They are generally considered to have fewer side effects than systemic agents, supporting exploration as long-term adjuncts; however, safety profiles vary by product and dose. Trials are generally small, methodologically heterogeneous, and short in follow-up, with sparse head-to-head comparisons and inconsistent dosing schemes. As a result, traditional pairwise meta-analyses are ill-suited for comprehensive, cross-supplement comparisons spanning disease severity, quality of life, biomarkers, and safety. Because most supplements have never been compared head-to-head in randomized trials, indirect evidence is essential for estimating their relative efficacy. Network meta-analysis (NMA) can formally integrate these indirect comparisons to generate clinically relevant contrasts (e.g., A–B and B–C informing A–C) that cannot be obtained from pairwise meta-analysis alone. Our NMA compares seven supplements across multidomain outcomes, reports effect sizes with CIs alongside SUCRA, and appraises certainty, thereby providing an integrated cross-supplement synthesis.

To address these gaps, we conduct a NMA that integrates direct and indirect evidence to estimate all pairwise contrasts within a single coherent framework. Under the transitivity assumption (comparability of populations, co-interventions, dosing/intensity, and outcome definitions across trials), indirect evidence (e.g., A–B and B–C) can inform missing comparisons (A–C), and we formally evaluate consistency between direct and indirect evidence.

This enables simultaneous, network-wide comparisons and explicit quantification of uncertainty where head-to-head data are absent or conflicting. We therefore report absolute effect sizes with 95% confidence intervals (CIs) alongside SUCRA rankings, emphasizing that rankings complement—but do not replace—clinically interpretable absolute effects. Prior evidence is suggestive yet heterogeneous for vitamin D (multiple RCTs (12–16)), omega-3 fatty acids (placebo-controlled RCT (17)), probiotics (clinical RCT (18)), curcumin (adjuvant RCTs with IL-22 reduction (19, 20)), XP-828L (two RCTs (21, 22)), and selenium (older or combination trials (23–26)).

To perform a NMA that compares and ranks the effectiveness and safety of adjunctive dietary supplements for plaque psoriasis, by combining direct and indirect evidence from Chinese- and English-language databases within a unified network, we aim to provide decision-relevant estimates and quantified uncertainty to inform—rather than determine—personalized treatment choices. Unlike earlier reviews limited to single supplements or pairwise comparisons, this study jointly evaluates multiple supplements and outcomes in a coherent framework.

The objective is to conduct a NMA comparing and ranking the effectiveness and safety of dietary supplements as adjunctive therapy for plaque psoriasis.

## 2 Methods

### 2.1 Registration

This systematic review and NMA were prospectively registered in the INPLASY register (Registration No. INPLASY202570119, DOI: 10.37766/inplasy2025.7.0119). Clinical trial number: Not applicable.

### 2.2 PICOS criteria

PICOS. Participants were adults with clinically confirmed plaque psoriasis. Interventions were adjunctive or standalone oral or topical dietary/nutritional supplements; comparators were placebo or standard therapy. Outcomes included PASI, DLQI, and adverse events. Only randomized controlled trials (RCTs) were eligible.

### 2.3 Literature search and data extraction

Two authors (P. D. C., J. Y.) independently searched eight databases—China National Knowledge Infrastructure (CNKI), VIP Chinese Science and Technology Journal Database, WanFang, Cochrane Library, PubMed, Embase, SinoMed, and Web of Science—from inception to 3 March 2025. Search terms included “psoriasis” and interventions (nutritional supplements, food supplements, herbal supplements, vitamins, folic acid, minerals, probiotics, fish oil, coenzyme Q10, amino acids, cherry extract, celery seed extract, and turmeric). Full search strategies are provided in Supplementary Table S1. Titles and abstracts were deduplicated in NoteExpress. Full texts were reviewed by D. C. and J. Y., with disputes resolved by Z. H. L. Extracted data included study details, participant characteristics, intervention specifics, risk of bias, and outcome data (Supplementary Table S7).

**Figure 1:**
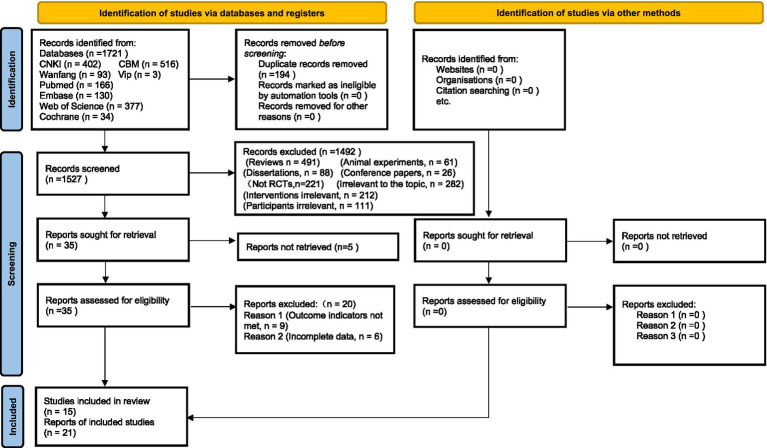
PRISMA 2020 study selection flowchart. This illustrates the selection process from 1,721 records to 21 RCTs, searched up to 3 March 2025.

Reporting. Reporting followed PRISMA 2020 and the PRISMA extension for NMA (PRISMA-NMA). The study selection process is shown in the PRISMA flow diagram (Figure 1), and the completed PRISMA-NMA checklist is provided in Supplementary Table S10; full search strategies are available in Supplementary Table S1.

### 2.4 Risk of bias assessment

The Cochrane Risk of Bias 2 (RoB 2) tool assessed five domains: randomization process, deviations from intended interventions, missing outcome data, outcome measurement, and selection of reported results. Each was rated low risk, some concerns, or high risk.

### 2.5 Statistical analyses

NMA was conducted within a frequentist framework using Stata 17.0. A random-effects consistency model was fitted via the network meta command, which automatically accounts for correlations in multi-arm trials when present; all studies included in this analysis were two-arm RCTs. Treatment ranking was obtained from the network rank function (5,000 iterations) to calculate the surface under the cumulative ranking curve (SUCRA). SUCRA represents the probability that a treatment ranks best or near-best across all interventions. Results are reported alongside absolute effect estimates and 95% CIs, noting that rankings are complementary summaries and do not imply clinical superiority.

Conventional pairwise meta-analyses were performed using random-effects models in Stata 11.0. Heterogeneity was quantified with I^2^ (<25% low, 25–50% moderate, >50% high); for completeness, the between-study variance (τ^2^) was also estimated. Because all three NMA outcomes (PASI, DLQI, and adverse events) formed star-shaped networks without any closed loops, inconsistency degrees of freedom were zero; therefore, neither global inconsistency (design-by-treatment Q statistic) nor local inconsistency (node splitting) could be statistically estimated. Subgroup analyses were prespecified by supplement class and treatment context (monotherapy vs. adjunctive regimens), and sensitivity analyses excluded studies with extreme dosing or high/unclear risk of bias. When I^2^ > 75% or inconsistency persisted, we reverted to random-effects pairwise meta-analysis and narrative synthesis.

Effect measures were risk ratios (RRs) for dichotomous outcomes and mean differences (MDs) for continuous outcomes, each with 95% CIs and a two-sided *α* = 0.05. For biomarkers measured on different scales, standardized mean differences (SMDs) were used; otherwise, MDs were reported. Publication bias was explored visually by funnel plots and, when ≥10 studies were available, statistically by Egger’s test (limited power acknowledged for fewer studies).

Study selection followed PRISMA 2020 (Figure 1). Certainty of evidence was appraised using GRADE, generally rated low to moderate because of substantial heterogeneity (e.g., *I*^2^ = 95.2% for PASI), imprecision (wide CIs), and small sample sizes (e.g., cytokine studies, *n* ≈ 60; Supplementary Table S8).

Extreme dosing was defined *a priori* as total vitamin D exposure exceeding approximately three times the network median (e.g., ≥10,000 IU/day equivalent) or employing non-standard intermittent bolus regimens such as monthly 100,000 IU. To support the transitivity assumption, we qualitatively examined key effect modifiers—baseline severity, concomitant NB-UVB or systemic therapies, dosing/intensity, and outcome definitions.

## 3 Results

### 3.1 Study characteristics

We identified 1,721 records from database searches up to 3 March 2025, removed 194 duplicates, and screened 1,527 titles and abstracts, excluding irrelevant studies (e.g., case reports, animal/cell studies, reviews, meta-analyses). Full-text review of 35 articles excluded non-RCTs, resulting in 21 RCTs (1,700 exclusions). Six additional articles from reference lists were included. A total of 1,463 patients (762 treatment, 701 control) with plaque psoriasis were analyzed (Supplementary Table S7). Selection adhered to PRISMA 2020 guidelines (Figure 1).

### 3.2 Risk of bias assessment

**Figure 2:**
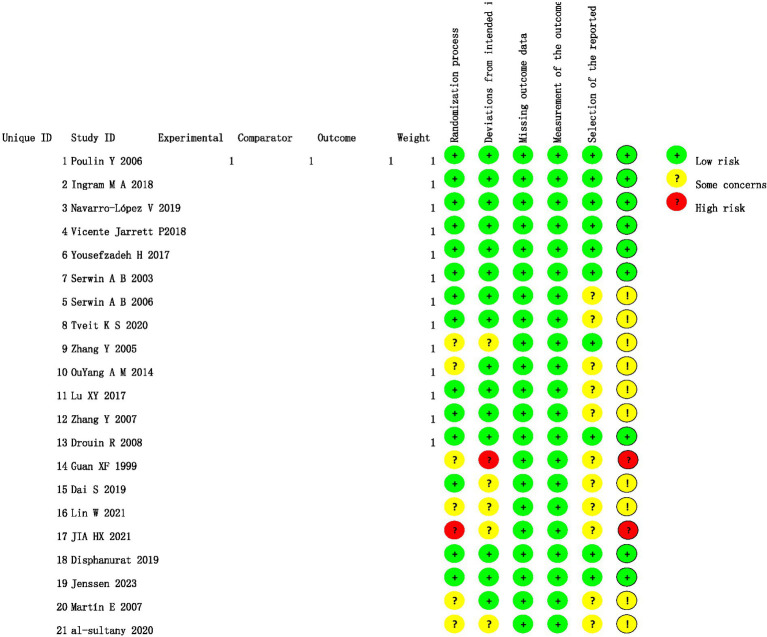
Risk of bias assessment for included studies. This summarizes RoB 2 across 21 RCTs for randomization, deviations, missing data, outcome measurement, and reported results. Colors: green (low risk), yellow (some concerns), red (high risk).

The Risk of Bias 2 (RoB 2) tool assessed 21 RCTs (Figure 2). Randomization: 11 studies (12, 13, 15, 17–19, 22, 26–29) used random tables or computerization (low risk); 2 (25, 30) used predictable methods (some concerns); 8 (14, 16, 21, 23, 24, 31–33) lacked details (high risk). Allocation concealment and blinding: 8 (12, 13, 15, 18, 22, 28–30) implemented measures or double-blinding (low risk); 5 (14, 16, 19, 32, 33) used open-label designs (some concerns or high risk). Evidence certainty was downgraded due to bias (Supplementary Table S8).

### 3.3 Evidence network

**Figure 3:**
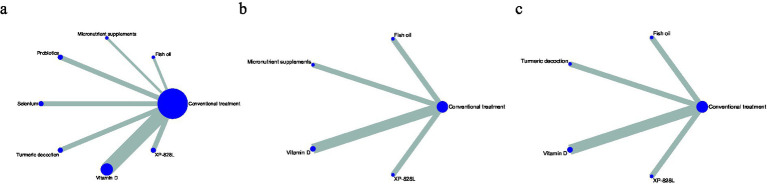
**(a–c)** Network geometry for each outcome. **(a)** PASI; **(b)** DLQI; **(c)** adverse events. Nodes represent interventions, and edges represent direct head-to-head comparisons. Node size is proportional to the total randomized sample, and edge width reflects the number of trials. The network was analyzed with a random-effects consistency model. PASI, Psoriasis Area and Severity Index; DLQI, Dermatology Life Quality Index; AEs, adverse events.

Therefore, a consistency model was applied for the NMA. Line thickness in the network plots reflects the number of direct comparisons, and node size represents the relative sample size. Because the networks contained no closed loops, inconsistency was not estimable and only heterogeneity could be assessed. Network plots for the Psoriasis Area and Severity Index (PASI), Dermatology Life Quality Index (DLQI), and adverse events are shown in Figures 3a–c.

### 3.4 PASI scores

Fifteen RCTs assessed seven dietary supplements for PASI in plaque psoriasis. NMA showed that vitamin D supplementation significantly reduced PASI scores versus conventional treatment (MD = −3.29, 95% CI − 6.38 to −0.20; SUCRA = 72.8%). Micronutrients ranked highest (SUCRA = 78.5%, MD = −4.97, 95% CI: −12.62 to 2.68), but wide CIs indicate low precision. Probiotics (SUCRA = 62.4%), curcumin (SUCRA = 46.3%), and XP-828L (SUCRA = 41.0%) showed moderate effects; selenium was lowest (SUCRA = 18.8%). Wide CIs warrant caution (Supplementary Tables S5a–c).

Conventional meta-analysis showed significant PASI improvement (MD = −1.95, 95% CI: −3.25 to −0.64, *p* < 0.001). Subgroup analysis of vitamin D with narrowband ultraviolet B (NB-UVB) versus NB-UVB alone indicated high heterogeneity (*I*^2^ = 95.2%, *p* < 0.001; MD = −12.40, 95% CI: −14.26 to −10.54, *p* < 0.001). Sensitivity analysis excluding seven studies (13, 14, 19, 22, 25, 26, 30) (due to variable doses/methods) reduced *I*^2^ to 47.7% (MD = −1.70, 95% CI: −2.28 to −1.12). Egger’s test (*t* = 0.03, *p* = 0.97) showed no publication bias (full results in Supplementary Table S9). Details are shown in Supplementary Figures S6a–g.

Network heterogeneity was high (I^2^ = 95.2%). This high heterogeneity reflects clinical and dosing variability rather than inconsistency, because inconsistency cannot arise in networks without closed loops. Therefore, inconsistency could not be assessed for PASI, and only heterogeneity was evaluated.

### 3.5 DLQI scores

**Figure 4:**
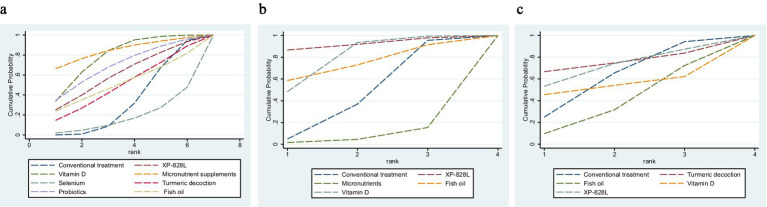
**(a–c)** Cumulative ranking curves and SUCRA values. **(a)** PASI; **(b)** DLQI; **(c)** adverse events. Curves show the cumulative probability that each intervention ranks at or above a given position in the network. Higher SUCRA values indicate a greater probability of being among the best treatments. Rankings are reported alongside absolute effect sizes in the main text and should not be interpreted as clinical superiority. PASI, Psoriasis Area and Severity Index; DLQI, Dermatology Life Quality Index; AEs, adverse events; SUCRA, surface under the cumulative ranking curve.

Five RCTs assessed XP-828L, micronutrients, fish oil, and vitamin D for DLQI. NMA found no significant reduction versus conventional treatment (Supplementary Table S5). XP-828L ranked highest (SUCRA = 85.2%, Figures 4a–c), but small sample sizes limit conclusions (Supplementary Table S5). Conventional meta-analysis showed a non-significant trend (MD = −0.47, 95% CI: −1.56 to 0.61, *p* = 0.203). Subgroup analysis indicated MD = −2.30 (95% CI: −5.68 to 1.08, *p* > 0.05), with XP-828L notable versus placebo. Egger’s test (*t* = −0.16, *p* = 0.880) and low heterogeneity (*I*^2^ = 32.8%) suggest reliable results (full results in Supplementary Table S9). Given small samples and overlapping CIs, DLQI findings—despite XP-828L’s favorable rank—should be interpreted cautiously pending larger confirmatory trials. As the DLQI network contained no closed loops, inconsistency could not be assessed, and only heterogeneity (I^2^ = 32.8%) was evaluated.

### 3.6 Pro-inflammatory cytokines (IL-6, IL-17, IL-23, IL-22)

Six RCTs (310 patients) assessed interleukin (IL)-6, IL-17, IL-23, tumor necrosis factor-alpha (TNF-*α*), soluble TNF receptor 1 (sTNFR1), and C-reactive protein (CRP). Random-effects meta-analysis showed a significant reduction (MD = −12.37, 95% CI: −14.90 to −9.84, *p* < 0.001). Subgroup analyses: vitamin D with NB-UVB reduced IL-6 (MD = −27.12, 95% CI: −30.61 to −23.61), IL-17 (MD = −29.34, 95% CI: −32.44 to −26.24), and IL-23 (MD = −75.34, 95% CI: −78.82 to −71.86); curcumin with conventional therapy reduced IL-22 (MD = −3.47, 95% CI: −5.03 to −1.91). Selenium and fish oil showed no effect on TNF-α or CRP (*p* > 0.05). Egger’s test (*t* = −2.43, *p* = 0.046) suggests possible publication bias. High heterogeneity (*I*^2^ = 99.7%) prompted sensitivity analysis; excluding (28, 29) (due to intervention/dose variability) reduced I^2^ to 47.6% (MD = 0.21, 95% CI: −0.01 to 0.42, *p* = 0.106, non-significant), indicating unstable overall effect (Supplementary Table S9). Meta-regression found no primary heterogeneity sources (*p* > 0.05). Given the extreme heterogeneity (I^2^ = 99.7%), likely reflecting differences in dosing and biomarker assays, these pooled results should be interpreted as exploratory. Detailed subgroup plots are shown in Supplementary Figures S6a–g.

### 3.7 Lymphocyte subsets (CD4, CD8, CD25)

One RCT (60 patients) assessed CD4^+^, CD8^+^, and CD25^+^ markers. The pooled estimate indicated a statistically significant effect (MD = 1.64, 95% CI 0.47–2.80), although the small sample size limits the certainty of this finding. Moderate heterogeneity was observed (I^2^ = 53.5%), prompting a sensitivity analysis. Subgroup analysis indicated that curcumin had no significant effect on CD4^+^ but increased CD8^+^ and CD25^+^ levels. Excluding heterogeneous CD8^+^ data (19)reduced I^2^ from 53.5 to 13.6% (MD = 1.23, 95% CI 0.18–2.29). Egger’s test (*p* = 0.929) suggested no publication bias. As this finding was derived from a single RCT with a modest sample size, the certainty of evidence remains low (Supplementary Table S9).

### 3.8 Physician global assessment scores

Two RCTs (138 patients) assessed physician global assessment (PGA) scores. The pooled analysis showed no significant difference between interventions (MD = −0.06, 95% CI − 0.41 to 0.29). Vitamin D was non-significant versus placebo, whereas XP-828L showed a borderline effect (MD = −0.26, 95% CI − 0.52 to 0.00). Substantial heterogeneity was observed (*I*^2^ = 85.7%), prompting a sensitivity analysis. We prespecified the exclusion of extreme-dosing outliers, removing the trial by Jarrett et al. (2018) (13)—which administered oral vitamin D₃ 100,000 IU monthly—a regimen distinct from the daily or weekly lower-dose oral schedules used in most other RCTs. This intermittent high-dose oral design may partly explain its outlier effect on heterogeneity—reduced I^2^ to 0% and yielded a consistent MD favoring XP-828L, although estimates remained imprecise. Small sample sizes limit generalizability (Supplementary Table S9). Because the PGA evidence network contained no closed loops, inconsistency could not be assessed, and only heterogeneity was evaluated.

### 3.9 Adverse events

Five RCTs assessed curcumin, probiotics, vitamin D, and XP-828L. Random-effects meta-analysis showed no significant difference (RR = 1.02, 95% CI: 0.94 to 1.10, *p* = 0.995). Subgroup analyses (all non-significant) suggest possible minor reductions with curcumin plus conventional therapy (*I*^2^ = 0%). Egger’s test (*p* = 0.402) showed no publication bias (Supplementary Table S9). Because the adverse-events network contained no closed loops, inconsistency testing was not applicable; only heterogeneity (I^2^ = 0%) was assessed.

### 3.10 Publication bias

**Figure 5:**
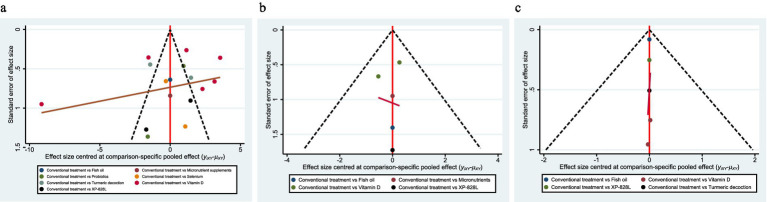
**(a–c)** Funnel plots for small-study effects. **(a)** PASI; **(b)** DLQI; **(c)** adverse events. Effect size is plotted against its standard error. Visual symmetry suggests no strong small-study effects, and formal Egger’s tests were non-significant where ≥10 studies were available. For outcomes with fewer studies, tests were underpowered. PASI, Psoriasis Area and Severity Index; DLQI, Dermatology Life Quality Index; AEs, adverse events.

Funnel plots (Figure 5a–c) for PASI, DLQI, and adverse events showed no apparent asymmetry, and Egger’s tests were non-significant for all major outcomes (*p* > 0.05), suggesting no strong small-study effects. The plots appear approximately symmetrical on visual inspection, supporting Egger’s test findings, although potential small-study effects cannot be entirely ruled out. For outcomes with < 10 studies (e.g., PGA, cytokines), formal bias testing was underpowered.

## 4 Discussion

### 4.1 Key findings

To our knowledge, this is the first NMA integrating Chinese and international RCTs to compare multiple dietary supplements across psoriasis outcomes. Prior reviews mainly assessed single supplements and did not provide cross-supplement comparisons spanning quality of life (QoL), biomarkers, and safety. Our NMA synthesized direct and indirect evidence in a unified framework, enabling comparative ranking across PASI, DLQI, PGA, cytokines (IL-6, IL-17, IL-22), and adverse events. Our findings are generally consistent with previous meta-analyses showing modest benefits of vitamin D and curcumin (5, 12, 34), but extend prior evidence by integrating multiple interventions and outcomes to inform personalized supplement strategies.

Because SUCRA reflects ranking probability rather than absolute efficacy, interpretation should rely on absolute effect estimates, precision, certainty, and safety—together with patient preferences—rather than rank probabilities alone. High SUCRA indicates a higher probability of ranking favorably but does not equate to clinically meaningful superiority; treatment choice should integrate absolute effects, safety, and patient preferences.

For PGA, vitamin D showed a non-significant trend. The wide CIs limit interpretability, suggesting that any potential benefit remains uncertain. XP-828L showed borderline improvement in DLQI/PGA after sensitivity analysis. Given the small sample sizes and overlapping CIs, these findings should be viewed as exploratory and require validation in larger trials. No supplement consistently changed all inflammatory markers or lymphocyte subsets, likely reflecting limited study numbers, heterogeneous dosing strategies, and the modest biologic potency of nutraceuticals relative to systemic therapies. Adverse events did not differ from controls overall (RR 1.02; low heterogeneity), with turmeric and XP-828L generally well tolerated. For PASI, the observed MDs suggest only modest clinical improvement and likely fall below conventional PASI50 thresholds. Given typical baseline PASI values in mild-to-moderate RCTs, an MD of −3.29 likely represents a modest improvement that falls below the conventional PASI50 response threshold. Future trials would benefit from reporting responder outcomes (PASI50/75/90) and DLQI MCID to better capture patient-relevant change. Estimates with wide CIs (e.g., micronutrients) should be viewed as exploratory. Our findings align with recent systematic reviews showing mixed or modest benefits, while extending the literature by integrating direct and indirect evidence across multiple outcomes, including biomarkers and safety. Clinically, vitamin D may be prioritized for severity/inflammation, XP-828L for QoL-focused symptoms, and curcumin where IL-22-linked inflammation is suspected; choices should consider comorbidities, tolerance, and patient preference.

Overall, while some supplements showed favorable ranking probabilities, absolute effect sizes were modest and clinically uncertain, underscoring the need for cautious interpretation.

From a clinical perspective, vitamin D, curcumin, and XP-828L appear the most promising based on mechanistic plausibility, consistency of direction, and favorable tolerability. In contrast, micronutrients and other interventions supported by sparse data or wide CIs should be considered not yet evidence-based and interpreted as exploratory pending further evaluation.

### 4.2 Limitations

Limitations include small sample sizes (only one RCT for cytokines, two for PGA, and fewer than 10 studies for most outcomes). Funnel plots appeared approximately symmetrical and Egger’s tests were non-significant, but small-study or publication bias cannot be excluded (Figures 5a–c). Heterogeneity was substantial for some outcomes (e.g., *I*^2^ = 85.7% for PGA; *I*^2^ = 53.5% for cytokines; and *I*^2^ = 99.7% for inflammatory factors) and decreased after sensitivity analyses (to *I*^2^ = 13.6 and 0%, respectively), likely due to differences in study design, populations, and dosing. In the vitamin D + NB-UVB subgroup, variability in vitamin D dosage, baseline serum 25(OH)D, and NB-UVB frequency likely contributed; excluding extreme regimens reduced I^2^, supporting this interpretation. Overall certainty was generally low to moderate due to heterogeneity, imprecision, and small samples, which constrains the strength of inferences.

### 4.3 Future directions

Vitamin D inhibits the Th17/IL-17/IL-23 pathway and regulates Treg/Th17 balance, particularly when combined with NB-UVB (34–36). Curcumin down-regulates NF-κB and IL-22 (15, 20). Omega-3–derived mediators may also attenuate inflammation through pro-resolving lipid pathways (37). These mechanisms are consistent with the observed reductions in IL-6, IL-17, IL-23, and IL-22, though biomarker shifts do not necessarily translate to clinical benefit and require confirmation in larger, adequately powered RCTs. XP-828L bovine whey protein extract / whey protein extract enhances epithelial barrier function and reduces neuroinflammation (38, 39). The gut–skin axis, influenced by microbiota and metabolic balance (12, 40), may also be modulated by XP-828L or turmeric-derived prebiotics (40). These complementary mechanisms suggest tailored therapeutic strategies for different clinical phenotypes. Large-scale RCTs are warranted. Future studies should standardize dosing and formulations and adopt core outcome sets (e.g., PASI75/90, DLQI MCID). Beyond methodological improvements, they should also extend follow-up, preregister protocols, and share data to improve evidence synthesis and translation. Future trials should also use standardized dosing regimens, clearly defined formulations, and adequate treatment durations to allow meaningful comparison across studies. Multi-center designs with longer follow-up periods and consistent reporting of PASI75/90 and DLQI MCID will be essential to establish reliable, evidence-based recommendations for supplement use in psoriasis.

## 5 Conclusion

No single dietary supplement optimally addresses all indicators in plaque psoriasis. This NMA (21 RCTs) highlights the differential roles of key supplements: Patterns observed across supplements suggest complementary roles: vitamin D appears more relevant to systemic inflammation, XP-828L to quality-of-life domains, and curcumin to IL-22-linked inflammatory pathways. These inferences remain tentative and require confirmation in future trials.

Personalization is advisable—vitamin D may be prioritized for severity/inflammation (especially with NB-UVB), XP-828L for QoL-centered symptoms, and curcumin where IL-22-linked inflammation is suspected. These patterns may reflect modulation of Th17/IL-23 and NF-κB-related pathways by vitamin D and curcumin, respectively; however, mechanistic inferences remain tentative.

High-quality, long-term RCTs are essential to confirm comparative effectiveness and guide personalized management.

## Data Availability

The original contributions presented in the study are included in the article/Supplementary material, further inquiries can be directed to the corresponding author.
