# Supplementary material for: Effectiveness and safety of dietary supplements in the adjunctive treatment of psoriasis: a systematic review and network meta-analysis
Source: Front Nutr. 2025 Dec 11;12:1718828. doi: 10.3389/fnut.2025.1718828 (PMC12738169; doi:10.3389/fnut.2025.1718828)
Supplement: Supplementary file 1 [file Data_Sheet_1.pdf]

Supplementary Material

Table of Contents

1 Supplementary Table S1. Search Strategy 2

2 Data Handling for Specific Outcomes..4

2.1 PASI Score Data Handling..4

2.2 DLQI Score Data Handling..4

2.3 Adverse events data handling..5

3 Primary and Secondary Outcomes 5

4 Sensitivity Analyses 5

5 Supplementary Table S5. League Tables 7

6 Supplementary Figure S6a – g. Subgroup meta-analyses by outcome..9

7 Supplementary Table S7. Baseline Characteristics and Additional Tables 10

8 Supplementary Table S8. Summary of evidence and certainty (GRADE) for primary and secondary outcomes..15

9 Supplementary Table S9. Sensitivity Analyses for Heterogeneity..16

10 Supplementary Table S10. PRISMA-NMA Checklist for Reporting Compliance..................17

1 Supplementary Table S1. Search Strategy

1 **Supplementary Table S1[1].** Electronic search strategies for the systematic review and network meta-analysis. Searches were conducted across eight databases—MEDLINE (PubMed), Embase, Cochrane CENTRAL, Web of Science, CNKI, Wanfang, VIP, and SinoMed—from inception to 3 March 2025. The search combined controlled vocabulary and free-text terms related to psoriasis, dietary supplements, and randomized controlled trials (e.g., “psoriasis,” “dietary supplement,” “randomized controlled trial”). Searches were limited to English and Chinese publications. The last searches for all databases were performed on 3 March 2025.

Database	Search Strategy
**PubMed**	("Dietary Supplements"[MeSH Terms] OR "Dietary Supplement"[Title/Abstract] OR "Supplements, Dietary"[Title/Abstract] OR "Dietary Supplementations"[Title/Abstract] OR "Supplementations, Dietary"[Title/Abstract] OR "Food Supplementations"[Title/Abstract] OR "Food Supplement"[Title/Abstract] OR "Food Supplements"[Title/Abstract] OR nutraceutical[Title/Abstract] OR herb supplement[Title/Abstract]) AND ("Psoriasis"[MeSH Terms] OR psoriasis[Title/Abstract] OR psoriases[Title/Abstract] OR "Pustulosis Palmaris et Plantaris"[Title/Abstract]) AND ("randomized controlled trial"[Publication Type] OR "controlled clinical trial"[Publication Type] OR randomized[Title/Abstract] OR randomised[Title/Abstract] OR placebo[Title/Abstract] OR "clinical trial"[Title/Abstract])
**Embase (Ovid)**	1. 'dietary supplement'/exp OR 'dietary supplement':ti,ab OR 'food supplement':ti,ab OR nutraceutical:ti,ab OR herb* supplement:ti,ab 2. 'psoriasis'/exp OR psoriasis:ti,ab OR psoriases:ti,ab OR 'pustulosis palmaris et plantaris':ti,ab 3. 1 AND 2 4. randomized controlled trial/exp OR 'controlled clinical trial'/exp 5. 3 AND 4

[1] *After the initial search, citations were imported into NoteExpress software for automatic deduplication. Two independent reviewers then manually checked and removed duplicates. In cases of multiple publications or overlapping data, the version with the most complete information was retained. Reference lists of included studies and relevant reviews were screened manually to identify potentially missed eligible studies.*

**Cochrane CENTRAL**	("Dietary Supplement":ti,ab,kw OR nutraceutical:ti,ab,kw OR "food supplement":ti,ab,kw OR "herbal supplement":ti,ab,kw) AND (psoriasis OR psoriases OR "pustulosis palmaris et plantaris"):ti,ab,kw
**Web of Science**	TS=("Dietary Supplement" OR "Dietary Supplementations" OR "Food Supplement*" OR nutraceutical OR herb supplement) AND TS=(psoriasis OR psoriases OR "Pustulosis Palmaris et Plantaris") AND TS=(random OR placebo* OR "controlled trial")
**WanFang**	主题: ("膳食补充剂" OR "营养补充剂" OR "食品补充剂" OR "草药补充剂" OR "维生素" OR "叶酸" OR "矿物质" OR "益生菌" OR "鱼油" OR "辅酶 Q10" OR "氨基酸" OR "樱桃提取物" OR "芹菜籽提取物" OR "姜黄")<br><br>AND 主题: ("银屑病")AND 全部字段: ("随机")
**VIP**	(M = ("膳食补充剂" OR "营养补充剂" OR "食品补充剂" OR "草药补充剂" OR "维生素" OR "叶酸" OR "矿物质" OR "益生菌" OR "鱼油" OR "辅酶 Q10" OR "氨基酸" OR "樱桃提取物" OR "芹菜籽提取物" OR "姜黄")<br><br>OR K = (同上))AND (M = ("银屑病") OR K = ("银屑病"))AND U = ("随机对照试验")
**CBM (SinoMed)**	#1 ("膳食补充剂" OR "营养补充剂" OR "食品补充剂" OR "草药补充剂 " OR "维生素" OR "叶酸" OR "矿物质" OR "益生菌" OR "鱼油" OR " 辅酶 Q10" OR "氨基酸" OR "樱桃提取物" OR "芹菜籽提取物" OR "姜黄")<br><br>#2 ("银屑病")<br><br>#3 ("随机" OR "随机对照试验")<br><br>#4 #1 AND #2 AND #3

**CNKI**	SU = ("膳食补充剂" OR "营养补充剂" OR "食品补充剂" OR "草药补充剂" OR "维生素" OR "叶酸" OR "矿物质" OR "益生菌" OR "鱼油" OR "辅酶 Q10" OR "氨基酸" OR "樱桃提取物" OR "芹菜籽提取物" OR "姜黄")<br><br>AND SU = ("银屑病")AND FT = ("随机")

2 Data Handling for Specific Outcomes

2.1 PASI Score Data Handling

Jarrett et al. reported only post-treatment Psoriasis Area and Severity Index (PASI) mean and its 95% confidence interval (CI). Standard deviations (SDs) were back-calculated from CIs and sample sizes using methods recommended in the Cochrane Handbook for Systematic Reviews of Interventions, Chapter 6.

Poulin et al. conducted a per-protocol analysis using PASI mean ± SD values for 36 participants in the treatment group and 37 in the control group. Four patients withdrew during the study.

Yousefzadeh et al. did not report PASI mean ± SD directly. Individual patient data were extracted from Table 2 to calculate: Treatment group: 5.13 ± 2.26 (n=15) Control group: 10.10 ± 2.35 (n=15)

Serwin et al. provided 4-week PASI scores as: Selenium group: 4.3 ± 3.92 (n=11) Placebo group: 1.67 ± 1.17 (n=11) Data were derived from Figure 1 and the main text.

Navarro-López et al. reported PASI scores graphically in Figure 2A, where error bars reflected ±SD. The extracted values were: Treatment group: 5.3 ± 2.2 (n=45) Control group: 6.7 ± 2.2 (n=43)

2.2 DLQI Score Data Handling

Jarrett et al. reported only post-treatment Dermatology Life Quality Index (DLQI) means and 95% CIs:
Treatment group (Vitamin D): 2.0 [0.5, 3.4]; Control group (Placebo): 2.5 [1.4, 3.6].
Standard deviations (SDs) were estimated from the CIs as approximately 3.36 and 3.53, respectively, using standard formulas (Cochrane Handbook, Chapter 6).

2.3 Adverse events data handling

Jarrett et al. reported mild adverse events in both the Vitamin D and placebo groups, including skin dryness, hyperlipidemia, and mild elevations in liver enzymes.
No serious adverse events or treatment discontinuations were observed.
Disphanurat et al. reported:
Treatment group (Vitamin D): 1 case of drowsiness and 1 case of nausea;
Control group (Placebo): 1 case of nausea.

3 Primary and Secondary Outcomes

Effect measures: Mean difference (MD) or standardized mean difference (SMD) for continuous outcomes (PASI, DLQI); risk ratio (RR) for adverse events, all with 95% confidence intervals (CIs). Beneficial direction: MD < 0 favours the intervention for PASI/DLQI; RR < 1 favours safety for adverse events.

Supplementary Figure S3a–c. Forest plots of (a) PASI, (b) DLQI, and (c) adverse events showing mean differences (MD) or risk ratios (RR) with 95% confidence intervals (CIs). Diamonds represent pooled effects.

(a) (b) (c)

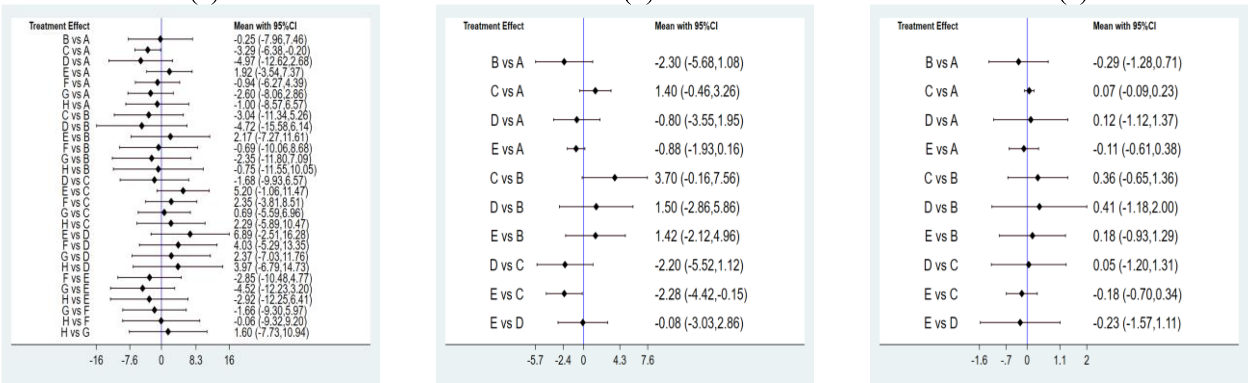

4 Sensitivity Analyses

To explore sources of heterogeneity, sensitivity analyses were conducted by sequentially excluding studies that differed markedly in intervention dose, treatment protocol, or outcome measurement scale. Specifically, high-dose vitamin D regimens (≥50,000 IU/month or single bolus ≥100,000 IU), non-

comparable NB-UVB protocols, and studies using biomarker assays on non-equivalent scales were examined separately. These exclusions reduced I^2 and improved model robustness.

Results of the leave-one-out sensitivity analyses for the main outcomes are presented in **Supplementary Figure S4a–e:**

Supplementary Figure S4a–e[2] (a) PASI including all studies (high heterogeneity);(b) PASI after excluding high-heterogeneity studies;(c) cytokines;(d) inflammatory markers (e.g., CRP, sTNF-R1);(e) Physician's Global Assessment (PGA).Each plot displays the pooled estimate and 95% confidence interval when one study at a time was omitted from the meta-analysis.

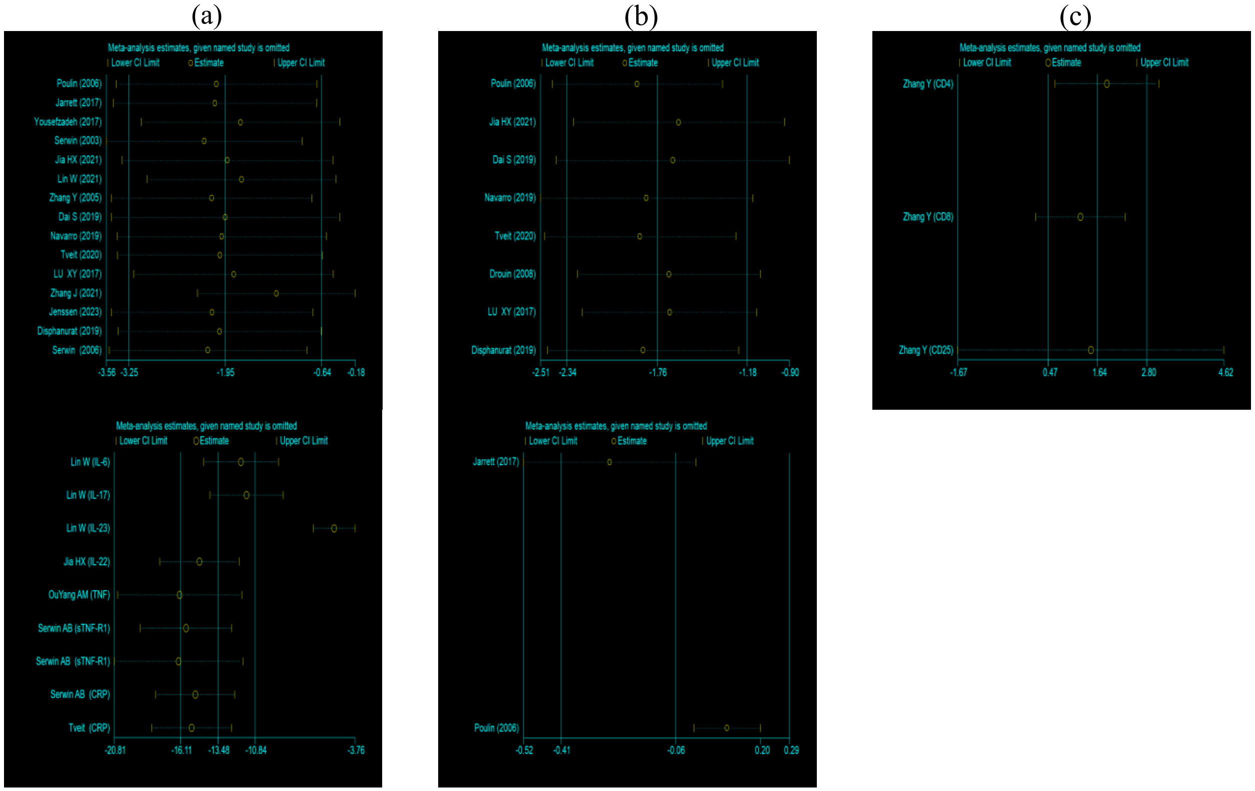

---

[2] *Leave-one-out sensitivity analyses for key outcomes of the network meta-analysis.*

(a) PASI (all studies); (b) PASI (after exclusion of high-heterogeneity studies); (c) cytokines; (d) inflammatory markers; (e) PGA.

Sequential omission of individual studies confirmed that no single trial disproportionately influenced the overall pooled estimates.

(d) (e)

5 Supplementary Table S5. League Tables

League tables for each outcome present relative treatment effects with 95% confidence intervals (CIs) for all pairwise comparisons in the network. Each cell shows the effect of the treatment listed in the row versus the treatment listed in the column.
For PASI and DLQI, negative mean differences (MDs) favour the treatment in the row; for adverse events, relative risks (RRs) less than 1 favour the treatment in the row. Supplementary Table S5a–c. League tables for pairwise network comparisons across outcomes.

Supplementary Table S5a[3].

**Micronutrients**							
-1.68(-9.93,6.57)	**Vitamin D**						
-2.37(-11.76,7.03)	-0.69(-6.96,5.59)	**Probiotics**					
-3.97(-14.73,6.79)	-2.29(-10.47,5.89)	-1.60(-10.94,7.73)	**Fish oil**				
-4.03(-13.35,5.29)	-2.35(-8.51,3.81)	-1.66(-9.30,5.97)	-0.06(-9.32,9.20)	**Turmeric decoction**			
-4.72(-15.58,6.14)	-3.04(-11.34,5.26)	-2.35(-11.80,7.09)	-0.75(-11.55,10.05)	-0.69(-10.06,8.68)	**XP-828L**		
-4.97(-12.62,2.68)	**-3.29(-6.38,-0.20)**	-2.60(-8.06,2.86)	-1.00(-8.57,6.57)	-0.94(-6.27,4.39)	-0.25(-7.96,7.46)	**Conventional therapy**	
-6.89(-16.28,2.51)	-5.20(-11.47,1.06)	-4.52(-12.23,3.20)	-2.92(-12.25,6.41)	-2.85(-10.48,4.77)	-2.17(-11.61,7.27)	-1.92(-7.37,3.54)	**Selenium**

[3] *Row = comparator; Column = intervention. Values represent mean differences (MDs) with 95 % confidence intervals (CIs) for row minus column. MD < 0 favours the treatment in the row (i.e., indicates greater PASI improvement). Values in bold indicate statistically significant comparisons (95 % CI not including 0).*

Supplementary Table S5b[4].

**XP-828L**				
-1.42(-4.96,2.12)	**Vitamin D**			
-1.50(-5.86,2.86)	-0.08(-3.03,2.86)	**Fish oil**		
-2.30(-5.68,1.08)	-0.88(-1.93,0.16)	-0.80(-3.55,1.95)	**Conventional therapy**	
-3.70(-7.56,0.16)	-2.28(-4.42,-0.15)	-2.20(-5.52,1.12)	-1.40(-3.26,0.46)	**Micronutrients**

Supplementary Table S5c[5].

**Turmeric decoction**				
**-0.18(-1.29,0.93)**	**XP-828L**			
**-0.29(-1.28,0.71)**	**-0.11(-0.61,0.38)**	**Conventional therapy**		
-0.41(-2.00,1.18)	-0.23(-1.57,1.11)	-0.12(-1.37,1.12)	**Vitamin D**	
**-0.36(-1.36,0.65)**	**-0.18(-0.70,0.34)**	**-0.07(-0.23,0.09)**	0.05(-1.20,1.31)	**Fish oil**

[4] *Row = comparator; Column = intervention. Values represent mean differences (MDs) with 95 % confidence intervals (CIs) for row minus column. Negative values indicate better quality of life (lower DLQI scores) for the treatment in the row. Values in bold indicate statistically significant differences (95 % CI not including 0).*

[5] Row = comparator; Column = intervention. Values represent relative risks (RR, 95 % CI) for row versus column. RR < 1 favours the treatment in the row (i.e., safer profile). Values in bold indicate statistically significant comparisons (95 % CI not including 1).

6 Supplementary Figure S6a–g. Subgroup meta-analyses by outcome.

Panels: (a) PASI subgroup analysis; (b) PASI subgroup analysis after exclusion of high-heterogeneity studies; (c) meta-analysis of inflammatory factors; (d) meta-analysis of cytokines; (e) meta-analysis of PGA; (f) subgroup analysis of adverse events; and (g) subgroup analysis of DLQI.

Squares represent individual study estimates (with size proportional to study weight), horizontal lines indicate 95% confidence intervals (CIs), and diamonds represent pooled effects.

I² and P values denote between-study heterogeneity.

Supplementary Figure S6a–g.

(a)

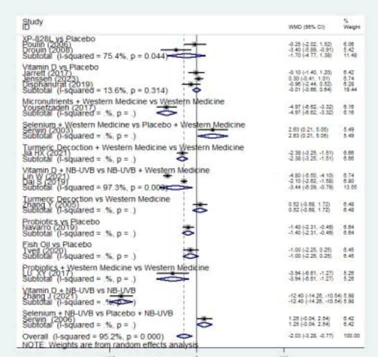

(b)

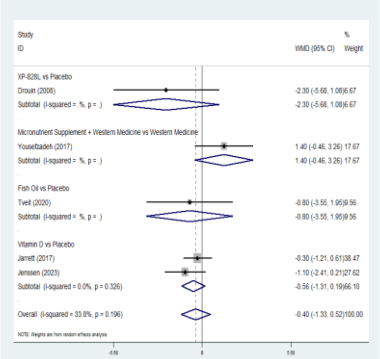

(c)

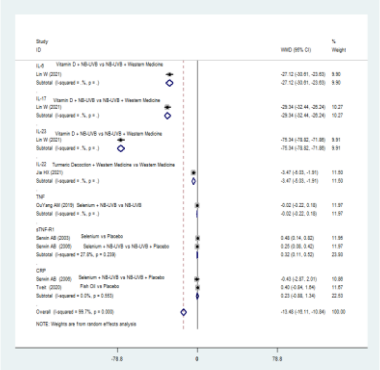

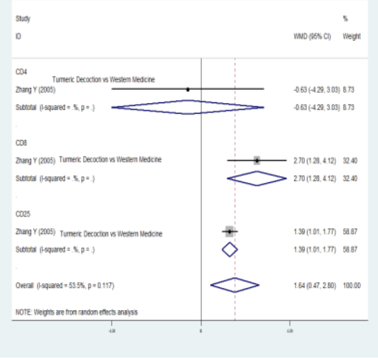

(d)

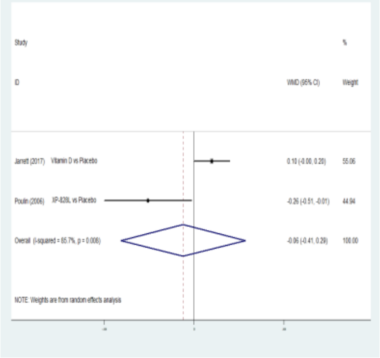

(e)

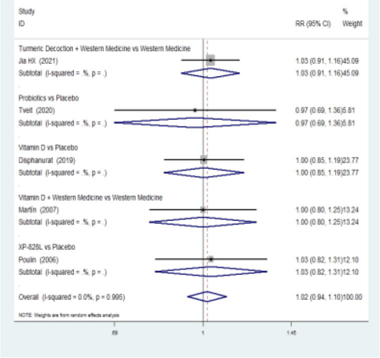

(f)

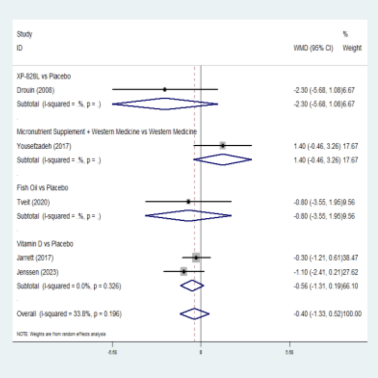

(g)

7 Supplementary Table S7. Baseline Characteristics and Additional Tables

Supplementary Table S7. Baseline characteristics of included studies.

Outcomes are coded as a–g (see footnote for details)

7 Supplementary Table S7[6].

Study	Country	Diagnostic Criteria	Participant Type	Sample Size (n)	Age (years)	Intervention	Duration	Administration	Outcomes
Poulin 2006	Canada	Inclusion: Clinically diagnosed stable plaque psoriasis; skin lesions covering ≥4% of body surface area (BSA); age ≥18 years. Exclusion: Pustular, erythrodermic, or palmoplantar psoriasis; active psoriatic arthritis; pregnant or breastfeeding women; other skin conditions that may interfere with evaluation; recent use of systemic medications (e.g., methotrexate, phototherapy) or potent topical corticosteroids.	Patients with mild to moderate psoriasis	42 Male: 13, Female: 29	T: 46.5±13.4, C: 47.1±12.6	XP-828L 5 g daily	16 weeks	Orally	a, b, d
Jarrett 2018	New Zealand	Inclusion: Aged 50–84 years; previously diagnosed with psoriasis; had psoriasis in the past 12 months; able to provide informed consent. Exclusion: Severe comorbidities.	Patients with mild psoriasis	23 Male: 15, Female: 8	T: 66.0±8.0, C: 66.0±8.0	Monthly supplementation with 100,000 IU vitamin D	48 weeks	Orally	a, b, c

[6] *Data are presented as mean ± standard deviation (SD) unless otherwise indicated. NA = not available. PASI = Psoriasis Area and Severity Index; DLQI = Dermatology Life Quality Index; AEs = adverse events. Active vitamin D (alfacalcidol/calcitriol) and cholecalciferol were analyzed as separate nodes in the network meta-analysis. Studies with ⩽4 weeks duration were included in sensitivity analyses only.*

Abbreviations: a, PASI score (Psoriasis Area and Severity Index); b, Physician's Global Assessment (PGA); c, Quality of Life score (e.g., DLQI); d, Adverse events; e, Inflammatory markers (e.g., sTNF-R1, CRP, IL-17A); f, Treatment efficacy rate; g, Cytokines (e.g., CD4, CD8, CD25)

Studies are identified by author and year to avoid confusion if reference numbers are renumbered during typesetting

Study	Country	Diagnostic Criteria	Participant Type	Sample Size (n)	Age (years)	Intervention	Duration	Administration	Outcomes
Ingram 2018	New Zealand	Inclusion: Age ≥18 years; diagnosed with chronic plaque psoriasis; mild, moderate, or severe. Exclusion: Severe kidney or liver disease; smoking; taking vitamin D >1000 IU/day.	Patients with chronic plaque psoriasis	67 Male: 39, Female: 28	T: 50.7±13.4, C: 46.7±13.7	Vitamin D (200,000 IU initially, followed by 100,000 IU monthly)	48 weeks	Orally	a
Yousefzadeh 2017	Iran	Inclusion: Chronic plaque psoriasis; skin lesions covering ≤20% BSA; no systemic treatment in the past 2 months. Exclusion: Type 1 diabetes, cardiovascular disease, liver dysfunction, pregnant or breastfeeding women.	Patients with chronic plaque psoriasis	15 Male: 9, Female: 6	38.53±11.05	Methotrexate + micronutrients (vitamin D, E, selenium, coenzyme Q10, B12)	12 weeks	Orally	a, c
Serwin 2003	Poland	Inclusion: Active plaque psoriasis; no systemic diseases.	Patients with plaque psoriasis	11 Male: 7, Female: 4	T: 31.0±11.03, C: 31.1±11.24	Selenium 200 µg/day + topical salicylic acid and dithranol	4 weeks	Oral and topical	a, e
Serwin 2006	Poland	Inclusion: Aged 18–50, chronic plaque psoriasis, skin phototype II or III, non-smokers. Exclusion: Recent selenium supplementation or comorbid diseases.	Patients with chronic plaque psoriasis	19 Male: 11, Female: 8	T: 31.26±10.49, C: 31.69±10.66	Selenium 200 µg/day + NB-UVB	4 weeks	Oral + Phototherapy	a, e
Tveit 2020	Norway	Inclusion: Stable plaque psoriasis, PASI <10, condition stable for at least 6 months. Exclusion: Use of ω3 fatty acids, cod liver oil, or other supplements.	Patients with plaque psoriasis	32 Male: 17, Female: 15	T: 47.0±12.8, C: 51.0±14.2	Herring roe oil capsules (10/day)	24 weeks	Orally	a, c, d, e

Study	Country	Diagnostic Criteria	Participant Type	Sample Size (n)	Age (years)	Intervention	Duration	Administration	Outcomes
Navarro-López 2019	Spain	Inclusion: Age 18–70 years; diagnosed with plaque psoriasis for at least 1 year; mild to moderate disease (PASI >6). Exclusion: Use of systemic treatments or antibiotics within the last 3 months.	Patients with plaque psoriasis	51 Male: 28, Female: 23	T: 41.57±13.23, C: 43.09±10.32	Probiotics + topical corticosteroids	12 weeks	Oral + Topical	a, b
Drouin 2008	Canada	Inclusion: Stable plaque psoriasis with PASI ≥4 and lesions involving 4%–15% of BSA. Exclusion: Pustular, erythrodermic psoriasis, active psoriatic arthritis.	Patients with mild to moderate psoriasis	16 Male: 12, Female: 4	T: 45.3±13.9, C: 55.2±13.9	XP-828L 800 mg/day	8 weeks	Orally	a, c
Dai 2019	China	Inclusion: Mild to moderate chronic plaque psoriasis, PASI <10, skin lesions <10% BSA. Exclusion: Severe infections or immune diseases.	Patients with mild to moderate psoriasis	30 Male: 30 Female: NA	46.8±9.7	NB-UVB + Vitamin D (800 IU/day)	12 weeks	Oral + Phototherapy	a, f
Lu XY 2017	China	Inclusion: Chronic plaque psoriasis, aged 18–70 years. Exclusion: Severe diseases or recent treatment.	Patients with chronic plaque psoriasis	25 Male: 20, Female: 5	T: 51.3±5.6, C: 52.2±5.9	Acitretin + probiotics	12 weeks	Orally	a, f
Zhuang J 2021	China	Inclusion: Chronic plaque psoriasis, no recent photochemotherapy or corticosteroid treatment. Exclusion: Other skin diseases or organ dysfunction.	Patients with plaque psoriasis	44 Male: 25, Female: 19	T: 48.82±5.31, C: 49.35±6.31	NB-UVB + Vitamin D (800 IU/day)	12 weeks	Oral + Phototherapy	a, f
Ouyang 2014	China	Inclusion: Chronic plaque psoriasis, no corticosteroid use in the last month.	Patients with plaque psoriasis	39 Male: 24,	T: 23.26±7.20, C: 26.21±6.12	NB-UVB + Selenomethionine	4 weeks	Oral + Phototherapy	e, f

Study	Country	Diagnostic Criteria	Participant Type	Sample Size (n)	Age (years)	Intervention	Duration	Administration	Outcomes
		Exclusion: Age <16, pregnancy.		Female: 15					
Zhang Y 2007	China	Inclusion: Chronic plaque psoriasis. Exclusion: Age <16, pregnancy, photosensitive diseases.	Patients with chronic plaque psoriasis	30 Male: 17, Female: 13	T: 36.3±9.6, C: 38.1±7.8	Turmeric decoction (twice daily)	4 weeks	Orally	a, g
Guan XF et al. 1999	China	Diagnosed with psoriasis vulgaris. Exclusion: None specified.	Patients with psoriasis	70 Male: 36, Female: 34	T: 36.2, C: 38	Indigo Naturalis Pills + sodium selenite	8 weeks	Orally	f
Lin W 2021	China	Inclusion: Chronic plaque psoriasis; normal liver/kidney function. Exclusion: Pregnancy, severe diseases.	Patients with chronic plaque psoriasis	50 Male: 23, Female: 27	T: 38.87±2.31, C: 39.02±2.23	Flumetasone + Vitamin D (0.25 µg, twice daily)	12 weeks	Oral + Topical	a, e
Jia HX 2021	China	Inclusion: Age 18–60, plaque psoriasis with skin lesions covering <20% BSA. Exclusion: Severe comorbidities or recent systemic therapy.	Patients with chronic plaque psoriasis	57 Male: 28, Female: 29	T: 38.91±5.19, C: 40.27±5.05	Calcipotriol + Turmeric decoction	10 weeks	Oral + Topical	a, d, f
Disphanurat 2019	Thailand	Inclusion: Age 18–70, mild psoriasis (PASI <10). Exclusion: Liver/kidney dysfunction, recent systemic therapy.	Patients with mild to moderate psoriasis	23 Male: 10, Female: 13	T: 52.39±14.19, C: 49.41±15.92	Vitamin D (60,000 IU every other week)	24 weeks	Orally	a
Jenssen 2023	Norway	Inclusion: Age 18–79, active plaque psoriasis (PASI >0), baseline 25(OH)D <24 ng/mL. Exclusion: Allergies, hyperparathyroidism, history of cancer.	Patients with plaque psoriasis	60 Male: 37, Female: 23	T: 53.3±10.9, C: 54.0±9.1	Vitamin D (100,000 IU loading, 20,000 IU weekly)	16 weeks	Orally	a, b, c

Study	Country	Diagnostic Criteria	Participant Type	Sample Size (n)	Age (years)	Intervention	Duration	Administration	Outcomes
Martín Ezquerra 2007	Spain	Inclusion: PASI 15–40, age >18. Exclusion: Contraindications to acitretin or calcitriol.	Patients with plaque psoriasis	20	Median age 61 (48–76)	Acitretin + Vitamin D (0.25 µg/day)	12 weeks	Orally	a, d
Al-Sultany 2020	Iraq	Inclusion: Age >18, diagnosed with chronic plaque psoriasis. Exclusion: Recent systemic therapy, severe comorbidities, pregnancy.	Patients with moderate-to-severe psoriasis	38 Male: 26, Female: 12	T: 36.54±4.7, C: 34.63±2.1	Topical corticosteroids + Vitamin D (50,000 IU/week)	12 weeks	Oral + Topical	a

8

Supplementary Table S8. Summary of evidence and certainty (GRADE) for primary and secondary outcomes.

Supplementary Table S8[7].

Outcome	Comparison	k	n	Effect (MD or RR [95% CI])	Certainty (GRADE)	Reasons for downgrade	Comments
PASI (MD)	Vitamin D vs Conventional care	5	356	MD −3.29 (−6.38 to −0.20)	●●○○ Low	Reasons: Very high inconsistency (I^2 95.5%), imprecision	Below/near the commonly reported PASI MCID threshold (≈3-5 points); interpret cautiously (see Carlin et al., 2004[8])
DLQI (MD)	XP-828L vs Conventional care	5	300	MD −0.47 (−1.56 to 0.61)	●●○○ Low	Imprecision, small sample	Overlapping CIs; interpret cautiously
Pro-inflammatory cytokines (MD)	Vit D + NB-UVB vs NB-UVB	6	310	MD −12.37 (−14.90 to −9.84)	●●○○ Low	Reasons: Extreme inconsistency (I^2 =	Effects not robust in sensitivity

[7] Abbreviations: PASI, Psoriasis Area and Severity Index; DLQI, Dermatology Life Quality Index; MD, mean difference; RR, risk ratio; CI, confidence interval; MCID, minimal clinically important difference; NB-UVB, narrowband ultraviolet B; AEs, adverse events. Evidence certainty was assessed using the GRADE approach considering risk of bias, inconsistency, indirectness, imprecision, and publication bias. Below PASI MCID = 3 (Smith et al., 2022); interpret cautiously. Overall, evidence certainty was low for PASI and DLQI, and high for adverse events. GRADE symbols: ●●●● = High; ●●●○ = Moderate; ●●○○ = Low; ●○○○ = Very low. Downgrade reasons: inconsistency (high heterogeneity), imprecision (wide CI or small n), indirectness, or risk of bias. Interpretation: MD < 0 or RR < 1 favours the intervention.

[8] *Carlin CS, Feldman SR, Krueger JG, Menter A, Krueger GG. A 50% reduction in the Psoriasis Area and Severity Index (PASI 50) is a clinically significant endpoint in the assessment of psoriasis. J Am Acad Dermatol. 2004;50(6):859-866. DOI: 10.1016/j.jaad.2003.09.014.*

Outcome	Comparison	k	n	Effect (MD or RR [95% CI])	Certainty (GRADE)	Reasons for downgrade	Comments
						99.7%), indirectness (assay variability)	
Adverse events (RR)	All supplements vs control	5	500	RR 1.02 (0.94–1.10)	●●●● High		No safety signal

9 Supplementary Table S9. Sensitivity Analyses for Heterogeneity

Supplementary Table S9[9].

Outcome	Primary analysis (MD/RR, 95% CI; I²)	Sensitivity analysis (after exclusion) (MD/RR, 95% CI; I²)	Studies excluded	Reason for exclusion	Interpretation
PASI (Vitamin D vs Conventional care, 8–24 weeks)	MD = −3.29 (95% CI −6.38 to −0.20); I² = 95.2 %	MD = −1.70 (95% CI −2.28 to −1.12); I² = 33.8%	7 studies [17, 21, 23–26, 28]	Bolus/high-dose vitamin D or heterogeneous NB-UVB protocols (≤ 4 weeks)	Effect attenuated; heterogeneity largely explained by dose/protocol differences
Pro-inflammatory cytokines (IL-6, IL-17, IL-23; pooled, exploratory)	MD = −12.37 (95% CI −14.90 to −9.84); I² = 99.7 %	MD = 0.21 (95% CI −0.01 to 0.42); I² = 47.6 %	2 studies [18, 19]	Non-comparable assays and dosing	Becomes non-significant after exclusions; exploratory due to assay/unit variability
PGA score	MD = −0.06 (95% CI −0.41 to 0.29); I² = 85.7 %	MD = −0.26 (95% CI −0.52 to 0.00); I² = 0 %	1 study [21] (Jarrett 2018)	Monthly 100 000 IU bolus (outlier)	Heterogeneity removed; Favours the intervention; CI upper limit = 0.00 indicates borderline significance (P ≈ 0.05); primary analysis non-significant (P > 0.05)
Adverse events	RR = 1.02 (95% CI 0.94 to 1.10); I² = 0 %	No material change (RR = 1.02 [95% CI 0.94–1.10]; I² = 0 %)	None	Not applicable	Robust finding

[9] *Abbreviations: PASI, Psoriasis Area and Severity Index; PGA, Physician's Global Assessment; NB-UVB, Narrowband Ultraviolet B; MD, mean difference; RR, risk ratio; CI, confidence interval. Sensitivity analyses were performed by sequentially excluding studies contributing to substantial heterogeneity ($I^2 > 90\%$) or methodological inconsistency. MD < 0 or RR < 1 favours the intervention. CI upper limit = 0.00 indicates borderline statistical significance (P ≈ 0.05); heterogeneity $I^2 = 0$ % after excluding Jarrett et al. (2018).*

10 Supplementary Table S10. PRISMA-NMA Checklist for Reporting Compliance

Supplementary Table S10[10].

Section/Topic	Item	Checklist Item	Reported on Page # or Section
**TITLE**			
Title	1	Identify the report as a systematic review incorporating a network meta-analysis (or related form of meta-analysis).	Page 1 (Title: "Effectiveness and Safety of Dietary Supplements in the Adjunctive Treatment of Psoriasis: A Systematic Review and Network Meta-analysis")
**ABSTRACT**			
Structured summary	2	Provide a structured summary including, as applicable: background; objectives; data sources; study eligibility criteria, participants, and interventions; study appraisal and synthesis methods; results; limitations; conclusions and implications of key findings; systematic review registration number.	Page 1 (Abstract section, including background, methods, results, conclusions)
**INTRODUCTION**			
Rationale	3	Describe the rationale for the review in the context of what is already known, including mention of why a network meta-analysis has been conducted.	Pages 1-2 (Introduction: Explains gaps in pairwise meta-analyses and rationale for NMA to compare multiple supplements)
Objectives	4	Provide an explicit statement of questions being addressed, with reference to participants, interventions, comparisons, outcomes, and study design (PICOS).	Page 2 (PICOS Criteria in Methods; Objectives stated in Introduction)
**METHODS**			

[10] *Abbreviations: NMA, Network Meta-Analysis; RCT, Randomized Controlled Trial; SUCRA, Surface Under the Cumulative Ranking Curve; PICOS, Participants, Interventions, Comparisons, Outcomes, Study design.*

Note: Checklist adapted from the PRISMA extension statement for reporting of systematic reviews incorporating network meta-analyses (Hutton et al., Ann Intern Med, 2015;162:777–784)

Section/Topic	Item	Checklist Item	Reported on Page # or Section
Protocol and registration	5	Indicate whether a review protocol exists and if and where it can be accessed (e.g., web address); and, if available, provide registration information, including registration number.	Page 2 (Methods: Registered in INPLASY, No. INPLASY202570119)
Eligibility criteria	6	Specify study characteristics (e.g., PICOS, length of follow-up) and report characteristics (e.g., years considered, language, publication status) used as criteria for eligibility, giving rationale. Clearly describe eligible treatments included in the treatment network, and note whether any have been clustered or merged into the same node (with justification).	Page 2 (PICOS Criteria: Adults with plaque psoriasis, RCTs, supplements alone or adjunctive; Exclusions listed)
Information sources	7	Describe all information sources (e.g., databases with dates of coverage, contact with study authors to identify additional studies) in the search and date last searched.	Page 2 (Literature Search: 8 databases from inception to March 3, 2025)
Search	8	Present full electronic search strategy for at least one database, including any limits used, such that it could be repeated.	Page 2 (Supplementary Table S1)
Study selection	9	State the process for selecting studies (i.e., screening, eligibility, included in systematic review, and, if applicable, included in the meta-analysis).	Page 2 (PRISMA flow diagram in Figure 1)
Data collection process	10	Describe method of data extraction from reports (e.g., piloted forms, independently, in duplicate) and any processes for obtaining and confirming data from investigators.	Page 2 (Two authors independently extracted data; Disputes resolved by third author)
Data items	11	List and define all variables for which data were sought (e.g., PICOS, funding sources) and any assumptions and simplifications made.	Page 2 (Extracted: Study details, participants, interventions, risk of bias, outcomes; Supplementary Table S7)
Geometry of the network	S1	Describe methods used to explore the geometry of the treatment network under study and potential biases related to it. This should include how the evidence base has been graphically summarized for presentation, and what characteristics were compiled and used to describe the evidence base to readers.	Page 3 (Evidence Network: Network plots in Figure 3a–c; Node size by sample, edge by trials)
Risk of bias within individual studies	12	Describe methods used for assessing risk of bias of individual studies (including specification of whether this was done at the	Page 2 (RoB 2 tool: Five domains rated low/some concerns/high risk; Figure 2)

Section/Topic	Item	Checklist Item	Reported on Page # or Section
		study or outcome level), and how this information is to be used in any data synthesis.	
Summary measures	13	State the principal summary measures (e.g., risk ratio, difference in means). Also describe the use of additional summary measures assessed, such as treatment rankings and surface under the cumulative ranking curve (SUCRA) values, as well as modified approaches used to present summary findings from meta-analyses.	Page 2-3 (MD for continuous, RR for dichotomous; SUCRA for rankings)
Planned methods of analysis	14	Prespecified subgroup analyses were performed according to supplement class and treatment context. Treatment ranking was performed using SUCRA. Because all networks were star-shaped and contained no closed loops, inconsistency could not be assessed. When heterogeneity remained very high ($I^2 > 75\%$), analyses reverted to random-effects pairwise meta-analysis or narrative synthesis.	Page 3 (Frequentist NMA in Stata 17.0; Random-effects consistency model; Inconsistency not assessable)
Assessment of Inconsistency	S2	All networks were star-shaped without closed loops; therefore, inconsistency (global or local) could not be assessed, and design-by-treatment or node-splitting methods were not applicable.	Page 3 (Inconsistency not assessable due to no closed loops)
Risk of bias across studies	15	Specify any assessment of risk of bias that may affect the cumulative evidence (e.g., publication bias, selective reporting within studies).	Page 3 (Funnel plots, Egger's test; Figure 5a–c)
Additional analyses	16	Describe methods of additional analyses if done, indicating which were pre-specified. This may include, but not be limited to, the following: Sensitivity or subgroup analyses; Meta-regression; Alternative formulations of the treatment network; Use of alternative prior distributions for Bayesian analyses (if applicable).	Page 3 (Subgroup by supplement class/treatment context; Sensitivity excluding extreme dosing/high bias; Meta-regression for heterogeneity)
**RESULTS**			
Study selection	17	Give numbers of studies screened, assessed for eligibility, and included in the review, with reasons for exclusions at each stage, ideally with a flow diagram.	Page 3 (PRISMA flowchart in Figure 1; 1,721 records to 21 RCTs)

Section/Topic	Item	Checklist Item	Reported on Page # or Section
Presentation of network structure	S3	Provide a network plot of the included studies to enable visualization of the geometry of the treatment network.	Page 3 (Figure 3a–c for PASI, DLQI, AEs)
Summary of network geometry	S4	Provide a brief overview of characteristics of the treatment network. This may include commentary on the abundance of trials and randomized participants for the different interventions and pairwise comparisons in the network, gaps of evidence in the treatment network, and potential biases reflected by the network structure.	Page 3 (Network plots in Figure 3a–c; node size reflects sample; edge width reflects number of trials)
Study characteristics	18	For each study, present characteristics for which data were extracted (e.g., study size, PICOS, follow-up period) and provide the citations.	Page 3 (Table 1: Study characteristics, n=1,463 patients)
Risk of bias within studies	19	Present data on risk of bias of each study and, if available, any outcome level assessment.	Page 3 (Figure 2: RoB 2 summary; 11 low randomization, etc.)
Results of individual studies	20	For all outcomes considered (benefits or harms), present, for each study: 1) simple summary data for each intervention group, and 2) effect estimates and confidence intervals. Modified approaches may be needed to deal with information from larger networks.	Pages 3-5 (Results sections: PASI, DLQI, cytokines, etc., with MD/RR and CIs)
Synthesis of results	21	Present results of each meta-analysis done, including confidence/credible intervals. In larger networks, authors may focus on comparisons versus a particular comparator (e.g. placebo or standard care), with full findings presented in an appendix. League tables and forest plots may be considered to summarize pairwise comparisons. If additional summary measures were explored (such as treatment rankings), these should also be presented.	Pages 3–5 (NMA results with MD/RR, CIs, SUCRA; Figure 4 for rankings
Exploration for inconsistency	S5	Because all networks lacked closed loops, inconsistency could not be evaluated. No design-by-treatment or node-splitting statistics were generated.	Page 3 (Inconsistency not assessable due to no closed loops)
Risk of bias across studies	22	Present results of any assessment of risk of bias across studies for the evidence base being studied.	Page 5 (Publication bias: funnel plots symmetrical; Egger's P>0.05; Figure 5a–c)

Section/Topic	Item	Checklist Item	Reported on Page # or Section
Results of additional analyses	23	Give results of additional analyses, if done (e.g., sensitivity or subgroup analyses, meta-regression).	Pages 4-5 (Sensitivity: Reduced I^2 after exclusions; Subgroups for vitamin D + NB-UVB, curcumin)
**DISCUSSION**			
Summary of evidence	24	Summarize the main findings, including the strength of evidence for each main outcome; consider their relevance to key groups (e.g., healthcare providers, users, and policy-makers).	Page 5 (Key Findings: Modest benefits, low-moderate certainty; Clinical prioritization)
Limitations	25	Discuss limitations at study and outcome level (e.g., risk of bias), and at review level (e.g., incomplete retrieval of identified research, reporting bias). Conclusions should reflect the extent of evidence and uncertainties, and discuss the likely effects or consequences (such as the need for further research).	Page 5 (Limitations: Small samples, heterogeneity, bias; Certainty low-moderate)
Conclusions	26	Provide a general interpretation of the results in the context of other evidence, and implications for future research.	Page 6 (Conclusion: Differential roles of supplements; Need for larger RCTs)
**FUNDING**			
Funding	27	Describe sources of funding for the systematic review and other support (e.g., supply of data); role of funders for the systematic review. This should also include information regarding whether funding has been received from manufacturers of treatments in the network and/or whether some of the authors are content experts<br>with professional conflicts of interest related to treatments in the network.	Page 6 (Funding: Supported by the Heilongjiang Provincial Administration of Traditional Chinese Medicine (grant ZHY2025-020). The funder had no role in the study.)
